# Supplementary material for: Elevated Blood Alcohol Concentration Is Associated with Improved Clinical Outcomes of Intravenous Thrombolysis Treatment in Acute Ischemic Stroke Patients—A Retrospective Study
Source: J Clin Med. 2023 Mar 14;12(6):2238. doi: 10.3390/jcm12062238 (PMC10051873; doi:10.3390/jcm12062238)
Supplement: Supplementary file 1 [file jcm-12-02238-s001.zip › jcm-2217924-supplementary.pdf]

Supplementary Table S1. Association between baseline clinical data, laboratory parameters and short term outcomes in cases (alcohol group) and controls.

	**Good outcome**	**Unchanged state**	**Poor outcome**	**p**
Number of patients, n (%)	114	105	21	
Male, n (%)	97 (85)	90 (86)	17 (80)	0.855
Age (years), median (IQR)	58 (51-67)	63 (56-70)	64 (60-70)	**0.010**
Etanol level (‰), median (IQR)	1.57 (0.59-2.28)	0.59 (0.29-0.83)	0.60 (0.10-1.51)	**0.016**
Acute alcohol consumption, n (%)	49 (43)	8 (8)	3(14)	**<0.001**
Affected brain area, n (%)				
VBI	31 (27)	29 (28)	4 (19)	0.723
Dominant MCA area	30 (26)	21 (20)	6 (29)	
Non-dominant MCA area	53 (47)	55 (52)	11 (52)	
Cerebrovascular risk factors, n (%)				
Hypertension	73 (64)	87 (83)	19 (90)	**0.001**
Diabetes mellitus	16 (14)	25 (24)	6 (29)	0.106
Hyperlipidemia	55 (48)	42 (40)	7 (33)	0.294
Atrial fibrillation	16 (14)	8 (8)	3 (14)	0.300
Previous stroke	14 (12)	12 (11)	5 (24)	0.292
Previous myocardial infarction	8 (7)	14 (13)	3 (14)	0.259
Previous peripheral artery disease	3 (2.6)	2 (2)	0 (0)	0.702
Current smoker	63 (55)	46 (44)	11 (52)	0.232
BMI, median (IQR)	26.8 (23.8-30.9)	28.1 (23.4-30.8)	28.7 (23.2-33.3)	0.365
Admission antiplatelet therapy, n (%)	21 (18)	30 (29)	7 (33)	0.127
Admission anticoagulant drug, n (%)	6 (5)	3 (3)	3 (14)	0.089
Admission antidiabetic therapy, n (%)	11 (10)	17 (16)	7 (33)	**0.014**
Admission lipid lowering therapy, n (%)	14 (12)	15 (14)	7 (33)	**0.044**
Admission NIHSS, median (IQR)	9 (6-13)	6 (5-11)	10 (7-13)	**<0.001** **0.005**‡ **0.022**#
OTN, min, median (IQR)	135 (108-175)	140 (112-182)	138 (101-183)	0.234
DTN min, median (IQR)	43 (32-57)	42 (31-56)	42 (36-56)	0.798
Rt-PA dose, mg, median (IQR)	71.0 (60.0-86.0)	72.0 (60.0-82.5)	77.0 (55.0-82.0)	0.790
Laboratory parameters, median (IQR)				
Serum sodium (mmol/L)	139.0 (137.0-142.0)	139.0 (137.5-141.0)	139 (137.0-142.0)	0.956
Serum glucose (mmol/L)	6.0 (5.3-7.1)	6.3 (5.5-7.8)	6.5 (5.6-8.6)	0.079
Creatinine (µmol/L)	73.0 (65.8-90.0)	73.0 (63.3-88.8)	79 (64.0-103.0)	0.467
HsCRP (mg/L)	2.48 (1.18-4.93)	2.53 (1.31-5.10)	4.0 (1.84-15.7)	**0.049**
AST (U/L)	22.0 (17.0-30.0)	19.0 (15.0-23.0)	19.0 (15.0-24.0)	**0.004** **0.004**‡
ALT (U/L)	20.0 (14.0-34.3)	18.0 (13.0-24.0)	18.0 (11.5-24.3)	0.200
GGT (U/L)	50.0 (28.5-92.3)	31.0 (20.8-51.5)	37.0 (22.5-51.0)	**0.003** **0.003**‡

WBC (G/L)	8.0 (6.7-9.5)	8.3 (6.4-10.6)	8.6 (6.9-10.6)	0.378
MCV (fL)	90.9 (89.9-94.3)	89.1 (86.4-92.5)	89.3 (85.3-92.8)	0.362
Platelet count (G/L)	230.0 (184.0-253.0)	215.0 (183.0-262.0)	216.0 (198.5-262.5)	0.934
APTT	28.4±3.0	28.5±3.4	27.5±3.1	0.467
INR	0.96 (0.93-1.01)	0.97 (0.94-1.02)	0.96 (0.92-0.99)	0.370
Stroke etiology (TOAST), n (%)				
Large artery atherosclerosis	36 (33)	29 (29)	13 (65)	0.052
Cardioembolic	23 (21)	17 (17)	3 (15)	
Small-vessel occlusion	24 (21)	30 (30)	3 (15)	
Other/undeterminated	28 (25)	24 (24)	1 (5)	
Admission BP systolic (mmHg), mean±SD	160.6±26.2	172.1±26.0	182.8±25.0	**<0.001**<br>**0.004‡**<br>**0.001⁺**
Admission BP diastolis (mmHg), mean±SD	91.0±16.3	95.9±15.7	101.4±19.0	**0.011**<br>**0.024‡**
Imaging data, median (IQR)				
ASPECTS on admission	10 (9-10)	10 (9-10)	10 (8-10)	0.67
ASPECTS at 24 h	8 (6-10)	8 (7-10)	7 (5-9)	0.503
Alcohol consumption habits, n (%)				
Non-, mild drinker	46 (41)	63 (60)	15 (71)	**0.003**
Moderate, heavy drinker	67(59)	42 (40)	6 (29)	

Abbreviations: IQR, interquartile range; VBI, vertebrobasilar insufficiency; MCA, middle cerebral artery; BMI, body mass index; NIHSS, National Institutes of Health Stroke Scale; OTN, onset-to-needle time; DTN, door-to-needle time; rt-PA, recombinant tissue plasminogen activator; hsCRP, high-sensitive C-reactive protein; AST, aspartate aminotransferase; ALT, alanine aminotransferase; GGT, gamma-glutamyl tansferase; WBC, white blood cell count; MCV, mean cellular volume; APTT, activated partial thromboplastin time; INR, international normalized ratio; TOAST, Trial of ORG 10172 in Acute Stroke Treatment; BP, blood pressure; ASPECTS, The Alberta Stroke Program early CT score.

‡good outcome versus unchanged state (ANOVA, Bonferroni post-hoc-test or Kruskal-Wallis, Dunn's post-hoc-test).
#unchanged state versus poor outcome (Kruskal-Wallis, Dunn's post-hoc-test).
⁺good outcome versus poor outcome (ANOVA, Bonferroni post-hoc-test).

Supplementary Table S2. Association between baseline clinical data, laboratory parameters and long term outcomes in cases (alcohol group) and controls.

	**mRS 0-2**	**mRS 3-6**	**p**
Number of patients, n (%)	168	72	
Male, n (%)	148 (88)	56 (77)	**0.040**
Age (years), median (IQR)	60 (52-69)	63 (59-69)	**0.005**
Etanol level (‰), median (IQR)	1.53 (0.52-2.19)	0.74 (0.48-1.55)	0.201
Acute alcohol consumption, n (%)	52 (31)	6 (8)	**0.001**
Affected brain area, n (%)			
VBI	53 (32)	11 (15)	
Dominant MCA area	33 (20)	24 (33)	**0.011**
Non-dominant MCA area	83 (48)	37 (52)	
Cerebrovascular risk factors, n (%)			
Hypertension	114 (68)	63 (88)	**0.002**
Diabetes mellitus	26 (15)	21 (29)	**0.014**
Hyperlipidemia	74 (44)	30 (42)	0.733
Atrial fibrillation	20 (12)	7 (10)	0.609
Previous stroke	20 (12)	11 (15)	0.475
Previous myocardial infarction	15 (9)	10 (14)	0.153
Previous peripheral artery disease	3 (1.8)	2 (2.8)	0.638
Current smoker	87 (52)	33 (46)	0.398
BMI, median (IQR)	27.1 (23.8-30.9)	28.4 (23.6-31.5)	0.410
Admission antiplatelet therapy, n (%)	36 (21)	22 (31)	0.130
Admission anticoagulant drug, n (%)	7 (4)	5 (7)	0.354
Admission antidiabetic therapy, n (%)	20 (12)	13 (18)	0.131
Admission lipid lowering therapy, n (%)	23 (14)	13 (18)	0.386
Admission NIHSS, median (IQR)	6.0 (5.0-10.0)	11.0 (8.0-14.0)	**<0.001**
OTN, min, median (IQR)	137 (109-180)	137 (107-181)	0.722
DTN min, median (IQR)	42 (31-58)	44 (36-56)	0.258
Rt-PA dose, mg, median (IQR)	70.5 (60.0-85.3)	74.0 (60.0-81.5)	0.805
Laboratory parameters, median (IQR)			
Serum sodium (mmol/L)	139.0 (137.0-142.0)	139.0 (137.0-142.0)	0.313
Serum glucose (mmol/L)	6.0 (5.4-7.1)	6.5 (5.6-8.9)	**0.003**
Creatinine (µmol/L)	73.0 (65.0-89.8)	73.0 (62.8-92.5)	0.968
HsCRP (mg/L)	2.4 (1.2-4.2)	3.4 (1.6-10.6)	**0.003**
AST (U/L)	21.0 (16.0-27.0)	20.0 (15.0-24.0)	0.149
ALT (U/L)	19.0 (14.0-28.8)	18.0 (14.0-25.0)	0.139
GGT (U/L)	38.0 (35.0-73.0)	38.0 (24.3-64.8)	0.879
WBC (G/L)	8.0 (6.4-9.7)	8.8 (7.1-10.9)	**0.008**
MCV (fL)	89.8 (85.9-93.3)	89.3 (86.9-92.8)	0.958
Platelet count (G/L)	224.0 (184.0-252.0)	219.5 (182.5-264.3)	0.970
APTT	28.4±3.2	28.3±3.3	0.856
INR	0.96 (0.93-1.01)	0.97 (0.93-1.02)	0.387
Stroke etiology (TOAST), n (%)			
Large artery atherosclerosis	44 (2)	34 (48)	
Cardioembolic	31 (20)	12 (17)	**0.015**
Small-vessel occlusion	44 (27)	13 (19)	
Other/undetermined	42 (26)	11 (16)	
Admission BP systolic (mmHg), mean±SD	163.0±25.6	177.0±27.7	**0.0002**

Admission BP diastolic (mmHg), mean±SD	92.6±15.5	96.3±18.2	0.115
Imaging data, median (IQR)			
ASPECTS on admission	10 (9-10)	10 (9-10)	0.743
ASPECTS at 24 hours	8 (6-10)	8 (6-10)	0.909
Alcohol consumption habits, n (%)			
Non-, mild drinker	85 (51)	39 (54)	0.612
Moderate, heavy drinker	83 (49)	33 (46)	

Abbreviations: mRS, modified Rankin Scale; IQR, interquartile range; VBI, vertebrobasilar insufficiency; MCA, middle cerebral artery; BMI, body mass index; NIHSS, National Institutes of Health Stroke Scale; mRS: modified Rankin Scale; ONT, onset to needle time; DTN, door-to-needle time; rt-PA, recombinant tissue plasminogen activator; hsCRP, high-sensitive C-reactive protein; AST, aspartate aminotransferase; ALT, alanine aminotransferase; GGT, gamma-glutamyl tansferase; WBC, white blood cell count; MCV, mean cellular volume; APTT, activated partial thromboplastin time; INR, international normalized ratio; TOAST, Trial of ORG 10172 in Acute Stroke Treatment; BP, blood pressure; SD, standard deviation; ASPECTS, The Alberta Stroke Program early CT score.
